# Behavioral determinants of continued use of Islamic FinTech: The moderating role of service and system quality

**DOI:** 10.1371/journal.pone.0351401

**Published:** 2026-06-17

**Authors:** Ayman Abdalmajeed Alsmadi

**Affiliations:** Al Zaytoonah University of Jordan, Amman, Jordan; Chinese Culture University, TAIWAN

## Abstract

The purpose of this study is to investigate the determinants of continued use of Islamic FinTech services through an examination of how useful Islamic FinTech services appear, what others think about the user’s use of Islamic FinTech services, how confident the user feels when using these services, and how much the user trusts the service provider in relation to the user’s actual use. This study will examine the relationship between the user’s actual use and their behavioral intention to continue to use the service. Additionally, this study will examine the role that both the service quality and system quality play as moderators for post adoption behaviors. In order to complete this study, a cross sectional survey was used to gather data from 232 active users of Islamic FinTech services in Jordan. A series of previously validated likert scale items were used to measure the variables used in this study. PLS-SEM with SmartPLS 4 software was utilized to test both direct and moderator effects in the proposed research model. Bootstrapping techniques were applied in order to determine if the structural paths in the model were significantly different from zero at a 95% confidence level. The results indicated that the proposed model accounted for 63.4% of the variance in actual use (R² = .634). The findings show that all four constructs; perceived usefulness, subjective norms, self-efficacy and perceived trust, are positively related to the user’s actual use of Islamic FinTech services. Also, there is a positive association between actual use and behavioral intentions to continue use of Islamic FinTech services. System quality has a moderating effect on the relationship between actual use and continuance intention while service quality does not have a statistically significant moderating effect. This study is one of few studies examining post adoption behavior in the context of Islamic FinTech services within Jordan. The study combines multiple theoretical frameworks including cognitive theory, social influence theory, and trust theory and incorporates both service quality and system quality into a singular framework to examine continuance behavior of Islamic FinTech usage.

## 1. Introduction

Introduction of newer and more innovative technological developments has given unprecedented chances for business houses in revolutionizing the finance industry by embracing newer digital solutions. This technological revolution and revolutionized desires and needs of customers have greatly transformed traditional practice in the finance industry, towards establishing Financial Technology (Fintech). Besides increasing the level of efficiency and access in the delivery of financial services, Fintech has also played a role in establishing new methodologies through which value creation and needs of various stakeholders can be met [1]. In this revolutionized environment, Islamic FinTech has emerged into mainstream by integrating teachings of Sharia with newer technologies’ potential in fostering ethical, inclusive, and speedy solutions in the finance industry [2].

Islamic FinTech represents a contemporary corporate model of money transactions that takes place according to Islamic ethical principles and takes advantage of technological advancements. It meets Sharia law prohibitions of interest (riba), excessive uncertainty (gharar), and investment in prohibited (haram) enterprise, and meets Muslims’ financing needs according to their ethical principles [3]. The utilization of Sharia values combined with Fintech services provides service to neglected cohorts while enhancing the transparency, efficiency, and trustworthiness of the service [4]. The relevance of Islamic FinTech is not confined to Muslim countries, as it also reflects the developing global interest in ethical finance. The combination of Sharia compliance and modern technology has created innovations that enhance the availability of numerous financial products and services, including Islamic online banking, P2P crowdfunding, and blockchain solutions.

Upon adoption of Islamic Fintech services, this research concentrates on the use of the service over time. Although elements like self-efficacy and perceived usefulness are prominent within the technology adoption literature, not much is said within the context of sustaining use, continued behavioral intention, and the Islamic finance sector, which arguably is a gap in literature. Contextually, this research focuses on continued use providing this research a basis of distinction from other research which focuses on single use of the service and addresses a significant gap around Islamic Fintech.

Service quality also accounts as one of the significant drivers in customer repeat purchase and satisfaction in Islamic FinTech services. Superior service increases services’ credibility and perceived usefulness and thus affects users’ behavioral intentions in adopting and using Islamic FinTech solutions [5]. Perceived usefulness, as the extent that the user believes using a specific system will enhance his or her performance, stands as the most significant construct used in determining users’ adoption behavior. Compatibility between users’ needs in finance and preferences and Fintech solutions has implications in their total satisfaction and perceived usefulness [1].

Subjective norm, or social pressure that one should or should not do some action, also affects Islamic FinTech adoption. Social pressure in support through members of one’s household, members of one’s immediate or extended family, or social or religious influencers can facilitate users’ adoption behavior for Islamic FinTech services by increasing social acceptability and value held in the solutions [6]. Perceived belief in one’s power or ability to do some specific task, or self-efficacy, also increases the adoption and continued use of Islamic FinTech services. Users with greater levels of self-efficacy are actually likely to utilize Fintech solutions efficaciously and fearlessly and are thus likely to increase continued use [7].

Perceived trust is also significant in establishing the behavioural intention to use and sustain Islamic FinTech services. Perceived trust in the technology, in the service provider organization, and in technology development increases users’ perceptions of the honesty of financial transactions, playing a significant determinant of the success in mass user adoption of the services [8]. Behavioural intention, being the individual’s attitude toward doing something specific, is also a suitable predictor of use. Behavioural intention to sustain Islamic FinTech services is grounded on the integration among perceived usefulness, subjective norm, self-efficacy, and perceived trust, which in turn determine sustained services adoption [9].

Tech anxiety and lack of self-efficacy are closely related when people are trying to use a technology for the first time. In Islamic Fintech self-efficacy is also related to the ability to use technology to make Sharia-compliant decisions. In Islamic Fintech services, lack of self-efficacy is associated with an inability to make quick and Sharia-compliant decisions during financial transactions due to anxiety of the technology. In research conducted by [7], it is noted that people with high self-efficacy manage to reduce technology anxiety, explore other features and use the Fintech services repeatedly. Research has shown that people with high self-efficacy are able to use mobile technologies confidently. This is also the case in Islamic Fintech services. [10] states that system quality leads to better user experience, customer satisfaction and positive reinforcement for continued use across all platforms. For the customer, the Islamic FinTech services can be used and upgraded seamlessly if there’s an integrated system to service quality, system quality, perceived usefulness and a subjective norm. This information can be used to formulate policies aimed at the sustained use and effective adoption of Islamic FinTech Solutions [11].

This study creates original knowledge by focusing on an important research gap in Islamic FinTech. Most previous studies have looked at first time usage of Islamic FinTech. However, there is limited research examining why users continue to utilize Islamic FinTech services, especially in developing countries such as Jordan. By combining TPB and DOI and analyzing how service quality and system quality moderate the relationship among variables; this research expands upon earlier models of technology acceptance. The results not only confirm that perceived usefulness, social norms, self-efficacy, and trust are all significant factors affecting user intention to adopt Islamic FinTech services, but it identifies the moderation effect of system quality as being distinct from the moderation effect of service quality. Therefore, this study has created theoretical and practical knowledge to further our understanding of Islamic financial services and to provide a foundation for future innovations within the field of Islamic financial services. The study was conducted in Jordan because of its rapidly expanding digital financial environment and its increasingly popular FinTech services. As well, Jordan has many Islamic financial institutions which makes it a good location to study post-acceptance behaviors associated with Islamic FinTech.

This study intends to fill a significant gap in the current research by providing answers to the following three Research Questions (RQ), RQ1: To what extent will perceived useful, subjectives norms, self-efficacy, and trust perceptions have an effect on the real use of Islamic FinTech services?, RQ2: Does the real use of Islamic FinTech has an impact on the users’ behavioral intent to continue to use the services?, Q3: To what degree is service quality and system quality moderating variable to determine if there is a positive relationship between the real use of Islamic FinTech and the users’ behavioral intent to continue to use?

In order to provide an integrative and holistic explanation of the drivers of Islamic FinTech, this research intend to contribute to both practice and academic communities. The research results will be beneficial to all stakeholders involved with financial technology and Islamic Finance.

There are still many theoretical gaps within the rapidly increasing amount of literature that focus on Islamic Fintech adoption and post-adoptive continued use of Islamic Fintech. There are two main gaps identified from previous studies. Firstly, most previous studies focused mainly on the users’ initial intention to adopt Fintech or Islamic Fintech. The researchers rarely investigate post-adoption behaviors such as continued use. Secondly, because many past studies researched individual behavioral constructs separately, it created fragmented theories which did not account for the interactional influence of belief factors related to cognition, social norms and control-based influences on users’ continued use of Islamic Fintech. Finally, the role of service quality and system quality were theoretically inconsistent. Some studies assumed that service quality and system quality were directly influencing variables whereas other studies indicated that their influence could be dependent upon user’s experiences.

## 2. Theoretical framework and hypotheses

The Theory of Planned Behavior (TPB) builds on the TAM framework by introducing subjective norms and perceived behavioral control, which is similarly posited in this study through subjective norms and self-efficacy [12]. This means that a person’s behavioral intention will be shaped not just by their personal attitude towards the behavior but also by the social norms within their environment and their level of self-confidence regarding their ability to carry out the intention. This is particularly important in the context of Islamic FinTech adoption where societal involvement and self- efficacy are paramount [13]. Also, the Diffusion of Innovation (DOI) Theory sheds light on the processes and patterns responsible for the growth and spread of new technology and concepts within a society. This theory can also be used in the spread and acceptance of Islamic FinTech innovations suggesting that its acceptance is conditioned by the gap it bridges over the existing innovations, its relevance in terms of the culture and lifestyle of the intended users, and ease of adoption [14]. These theories complement each other so effectively that they provide sufficient coverage explaining the different aspects of relationships that affect the acceptance and maintenance of Islamic FinTech services with a focus on the modern sociotechnical environment [15].

Recent Islamic FinTech adoption studies were mostly focused on the individual characteristics of technology adoption. These include: perceived usefulness; perceived trust; social influence; and self-efficacy. The literature is fragmented and does not fully explain the way that all these factors are interrelated to generate continued usage behaviors in post-adoption environments. TAM based studies provide explanations of cognitive assessments including usefulness and ease of use. TPB oriented studies provide explanations of both normative assessment and behavioral control. DOI-based studies highlight the importance of innovation compatibility and perceived advantage. In spite of these contributions there have been few attempts at combining these perspectives into one post-adoption framework for Islamic FinTech services.

Additionally, the empirical results from previous studies show mixed support regarding the impact of quality related variables. For example, some studies posited that service quality and system quality would directly affect the level of continuance intent for users. Other studies proposed that the effect of quality related variables could be dependent upon prior experiences or usage behaviors of users. Given this mix of results it can be inferred that a more detailed explanation of how quality related variables function in a model of continuance behavior is required. In accordance with this inference, the current study frames service quality and system quality as moderators of continuance behaviors and not as direct antecedents of continuance behaviors, thereby providing an alternative view on how users’ experiences with Islamic FinTech platforms impact sustained behavioral intention.

Finally, many prior Islamic FinTech studies focused on the intention to adopt the technology initially and not actual post-adoption usage behaviors. As such, there exists a theoretical gap as well as a practical gap. Continuance intention is often affected by multiple aspects that accumulate over time, such as trust reinforcements through repeated interactions and system performance improvements, in contrast to only pre-adoption perception. Rather than simply treat prior findings as separate predictors of technology adoption, the current study combines cognitive, social, trust related, and quality related views into a single post-adoption framework for Islamic FinTech continuance behavior.

In addition to a user’s actual use being an action-based result of the interaction of their cognitive assessments of a product, social influences they perceive, their assessment of how capable they are to perform the desired behavior, and their trust in the institution providing it, Actual Use in TPB will occur only if there exist positive attitudes toward the behavior, subjective norms that support the behavior, and appropriate levels of perceived behavioral control. In the case of Islamic Fintech products, which operate within both ethical and regulatory constraints (e.g., Sharia compliant), trust in digital financial infrastructure adds another layer to this same process. Perceived Usefulness, Subjective Norms, Self-Efficacy and Perceived Trust are therefore conceptualized as complementary factors that can independently contribute to a user transitioning from a positive perception of a product to repeated and sustained use of the product. This establishes the basis on which these factors should be modeled as direct antecedents of actual use as opposed to merely predictors of intentions.

### 2.1 Perceived usefulness and actual use of Islamic FinTech

[5] stated, perceived usefulness has the highest importance for the actual use of Islamic FinTech as it affects user confidence and adoption intention. Based on [9], individuals who have a positive attitude towards Islamic FinTech from Sharia, convenience of doing business, and transparency will use their services. Likewise, [16] emphasized that Islamic FinTech applicable trust allows participants to be positive towards its usage in their financial status and position. [17] explained that perceived usefulness triggers a behavior intention, which is put into practice in real life. According to their research, users who see Islamic FinTech platforms as effective and reliable are from a non-use traditional bank. [18] also explained that greater perceived usefulness due to embracing digital technologies overcomes the barrier of access. Subject to the availability of digital efficiency, [11] emphasized that easy-to-use Islamic FinTech platforms further enhances the perception of usefulness. Conversely, [19] demonstrated that mobile technology has multi-facet advantages, such as greater acceptance and institutional embedding. In post-adoption contexts, users rely on experienced performance outcomes rather than expectations, meaning that technologies perceived as useful are more likely to be embedded into routine financial activities. In Islamic FinTech, this effect is further amplified by the dual nature of usefulness, which combines functional efficiency with Sharia compliance and ethical value. As users repeatedly experience these benefits, perceived usefulness evolves from a cognitive evaluation into a reinforcing driver of habitual usage behavior. Therefore, perceived usefulness is not only a determinant of intention but also a direct predictor of actual use, particularly when users have already engaged with the technology and recognized its practical and ethical advantages. Lastly, [20] emphasized that trust to regulators enhances Islamic FinTech’s perceived usefulness and yields greater actual use. Therefore, this study posits:

H1: Perceived Usefulness Significantly impacts Actual Use of Islamic FinTech.

### 2.2 Subjective norms and actual use of Islamic FinTech

Subjective norms describe the pressure and the social factors, which include family, friends, and even religious leaders, that can motivate or deter the adoption and use of a certain technology [21]. In Islamic finance, [13] observed that community subjective norms tend to dominate, especially in environments where a high value of religious and social capital is present. Their research showed that Islamic FinTech usage is more likely when people believe they are receiving strong social support from peers and family. Likewise, [22] cites the fact that Islamic FinTech is more widely used due to supportive word-of-mouth from reputable religious scholars. [23] noted how subjective norms influence behavioral intention, which further determines the actual use of a product. They reported that collectivist societies embraced Islam Fintech faster than others. This finding is in line [24], who argued that users of Islamic finance who are actively engaged in social circles that do not resist the use of technology are more likely to use these platforms. [20] examined how social media helps shape subjective norms where users can have conversations regarding the usefulness and trustworthiness of Islamic FinTech. In particular, their findings are consistent with [25], who argued that trust and social validation of these technologies as correct financial behaviors is fabricated through digital advocacy and community conversations. [22], focused on how government and institutional support has contributed to subjective norms, and suggested that state-sponsored Islamic FinTech projects within the Muslim context create additional public confidence and hence need greater acceptance. According to [26], community-based financial institutions advertising Islamic FinTech services also use religion as social capital, which increases positive subjective norms and motivates people to abandon traditional banking. Therefore, this study posits:

H2: Subjective norms Significantly impacts Actual Use of Islamic FinTech

### 2.3 Self-efficacy and actual use of Islamic FinTech

The perception of self-efficacy as one’s belief of their ability to utilize technology successfully influences the technological actual use of Islamic FinTech [15]. [27] showed that self-efficacious individuals tend to have more confidence when using Islamic FinTech platforms and thus, their adoption levels are higher. Likewise, [28] pointed out that users skilled in conducting digital transactions are more likely to make the switch from conventional Fintech services to Islamic ones. [29] argued that self-efficacy enhances positive intentions towards the behavior, which is using the service. Users who possess strong digital competencies are seemingly more willing to make use of the more sophisticated features of Fintech, as these users seem to have a reason to. Supporting this, [30] claimed that self-efficacy defeats technological anxiety and enables the user to engage more freely to Islamic FinTech applications. [31] contend that self-efficacy induced through specialized financial literacy programs makes people more likely to actually use the technology. Mobile friendly interfaces and customer care services also promote self-efficacy as noted by [32] thus, promoting utilization. [33] argued that self-efficacy enables users to have confidence in the security measures put in place by Fintech resources. On the other hand, [34] revealed the moderating role self-efficacy has on leveraging perceived ease of use on Islamic FinTech services. Therefore, this study posits:

H3: Self-efficacy Significantly impacts Actual Use of Islamic FinTech

### 2.4 Perceived trust and actual use of Islamic FinTech

[35] noted that customers are more willing to adopt Islamic FinTech platforms when they view them as trustworthy, particularly with regards to their adherence to Islamic finance principles and transparency. [36] argue that having trust in data security, fraud prevention, and ethical financial dealings makes users more confident and facilitates increased adoption. It was [37] contention that perceived trust has an influence on behavioral intention which in turn affects actual usage. Their analysis demonstrated that financial institutions with high levels of consumer trust stemming from strong protection policies and transparency in their dealings tend to have higher user engagement with Islamic FinTech. This shift to Islamic FinTech solutions from conventional banking was viewed as a positive change due to ease of technological adoption on these platforms due to reduced trust barriers, as noted by [38]. [39] observed that trust in a system is built with the existence of regulatory frameworks that ensure adherence to the Islamic financial laws and are deeply relied on by users. Regarding those who operate within the sphere of investment [40] states that wholesome customer experience and service delivery has a way of boosting trust in Islamic FinTech. Financial trust is an issue that [41] recognize as one that perceived trust solves. Islamic FinTech services are more trusted and relied upon when there is institutional reputation and credibility which [42] outlined as central to increased adoption and sustained usage of the service. While service quality and system quality are frequently examined as direct antecedents of technology adoption, post-adoption research suggests that their influence may be more context-dependent once users gain experience with a system. In continuance settings, actual use reflects an established behavioral pattern, whereas quality-related factors shape how this usage translates into long-term behavioral intention. Accordingly, service quality and system quality are conceptualized in this study as moderating conditions that strengthen or weaken the relationship between actual use and continuance intention. This positioning reflects their role as boundary mechanisms that condition sustained engagement rather than primary drivers of initial or repeated use. Therefore, this study posits:

H4: Perceived trust Significantly impacts Actual Use of Islamic FinTech

### 2.5 Actual use of Islamic FinTech, service quality and behavioral intention to continue use

The context of using Islamic FinTech and the willingness to persistently utilize it correlatively depends on service quality [43]. Quality services improve user satisfaction and ensure that onboarding the service turns into sustained usage on Islamic FinTech platforms [44]. [45] noted that Islamic FinTech is beneficial since its actual use cultivates users’ confidence which they have in their intent to continue using any of these services. On this aspect, their study also verified that customers have a level of service quality which influences this relationship, that is customers will be inclined to use Islamic FinTech services to the extent that their services are efficient, reliable and customer friendly. [46] showed that service quality (system service quality, responsiveness, ease of use) can moderate the relationship between user satisfaction and technology adoption in m-commerce in Jordan. This supports the idea that quality elements can strengthen or weaken adoption effects. [47] indicated that providing high quality basic services is essential towards achieving the desired behavioral intention. [48] summarized this effect on trust and stated that service quality improves trust, which enhances continued user engagement. According to [49], financial services that are tailored to fit user needs, along with excellent customer service, enhance behavioral intention. On the other hand, [50] argues that the degree to which one is willing to use Islamic FinTech is directly related to how fast and precise transactions are carried out. Finally, [18] argued that increased satisfaction will follow from high service quality. Therefore, this study posits:

H5: Service quality significantly moderates the relationship between Actual Use of Islamic FinTech and Behavioral Intention to Continue Use.

### 2.6 Actual use of Islamic FinTech, system quality and behavioral intention to continue use

System quality as a factor in sustaining the use of Islamic FinTech is especially important, considering that it directly affects both the actual use and the intention to continue using Islamic FinTech [51]. A dependable system boosts user satisfaction and guarantees that the user’s first experience translates into ongoing satisfaction with Islamic FinTech platforms [52]. Frequent users of Islamic FinTech services exhibit increased reliance on them, which boosts their intentions toward continued use [9]. Nonetheless, their research also showed that the strength of this relationship was moderated by system quality in terms of ease of use, reliability, and security, thus making continued use more probable when the platforms are operating well. [53] further noted that features of system quality, including fast processing speed, low response time, and easy to understand layout, create a positive relationship between use of the systems and continued participation. According to [11], the majority of users are more disposed to use Islamic FinTech services over and over again when the system is stable, responsive, and safe. These studies showed that factors like system performance, especially frequent technical errors and breaches of security greatly reduce the chances of users sustaining a desired behavior towards a system. However, [51] posited that achieving higher levels of system quality promotes trust and confidence, conditions that are critical for users’ long term adoption of the system. According to [28], users’ continuous engagement with Islamic FinTech platforms is more likely when the system performance remains stable and meets their expectations. [54] previously established that retention rates are correlated with technical efficiency and user experience. [55] pointed out that system quality increases digital trust. Therefore, this study posits:

H6: System quality significantly moderates the relationship between Actual Use of Islamic FinTech and Behavioral Intention to Continue Use.

### 2.7 Actual use of Islamic FinTech and behavioral intention to continue use

The majority of users possessing a favorable view of Islamic Finetnch platforms tend to remain active users of these financial technologies [56]. Active users of Islamic FinTech services have a strong usage intention because they consider it reliable service [26]. [57], on the other hand, illustrated that satisfaction with ease of transaction, convenience, Sharia compliance, and other factors that enhance service delivery satisfaction directly affect the behavioral intention towards the Islamic FinTech sector. The actual use of the platform makes it easier to trust the brand, which lowers skepticism and stimulates use [58,59]. Increased interaction with Islamic FinTech platforms and favorable perceptions of these services aid in viewing these services as fundamental financial instruments, which in turn increases the intention to use these services in the future. Smooth functionality, secure transactions and other positive aspects of digital interactions increases usage behavior [60]. Usage retention is explained by increases in service customization and financial literacy [61]. User satisfaction, especially from real-world application of technology, strengthens behavioral intention [62–64]. Therefore, this study posits:

H7: Actual Use of Islamic FinTech Significantly impacts Behavioral intention to continue use

This study is theoretically grounded in the integration of the Theory of Planned Behavior (TPB) and Diffusion of Innovation (DOI) theory to explain continued use behavior in Islamic FinTech contexts. TPB provides a robust foundation for understanding how cognitive beliefs (perceived usefulness and trust), normative pressures (subjective norms), and control beliefs (self-efficacy) translate into actual behavior. DOI complements this perspective by explaining how users evaluate the relative advantage and operational performance of technological innovations over time. By integrating these perspectives, the present framework moves beyond intention-based adoption models and focuses on post-adoption dynamics, where actual use becomes a pivotal mechanism linking behavioral beliefs to continuance intention. This theoretical integration enables a more comprehensive explanation of sustained Islamic FinTech usage rather than one-time adoption decisions.

This research extends the combination of the Theory of Planned Behavior (TPB) and Diffusion of Innovation (DOI) theories from initial technology adoptions to post-adoption continuance behaviors in Islamic FinTech applications. Unlike past studies which have generally limited their examination to behavioral intentions as the ultimate decision factor, this research positions actual usage as a primary behavioral mechanism through which beliefs, social influences, and trust and quality related factors are linked to continuance intentions. Therefore, it will provide an additional explanatory model for understanding how Islamic FinTech is used over time; whereas past models provided explanations for initial technology adoption choices.

Additionally, this research makes a theoretical contribution by combining cognitive based factors (perceived usefulness and trust), social based factors (subjective norms), behavioral control based factors (self-efficacy), and technological performance conditions (service quality and system quality) into a singular integrated model. Past research has generally investigated each construct separately using TPB or DOI based frameworks. The current research combines both sets of constructs in order to show how the joint effect of user experience and system performance influence continued use of Islamic FinTech services. Finally, the research also offers a new perspective on service quality and system quality by reconceptualizing them as moderators as opposed to being directly causal. This represents a significant contribution to the Islamic FinTech literature since it demonstrates that continued use of an Islamic FinTech application can be more dependent upon a consumer’s accumulation of knowledge about system reliability and operational performance than on consumers’ perceptions at the time of initial adoption.

**Fig 1:**
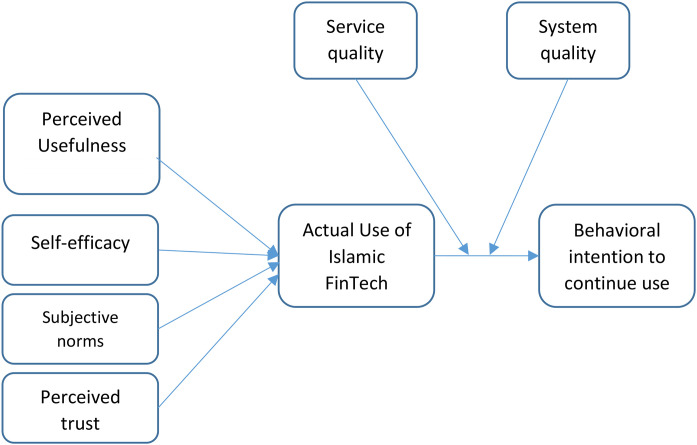
Research model.

Fig 1 presents the conceptual framework of this study. The model proposes that perceived usefulness, subjective norms, self-efficacy, and perceived trust act as key antecedents of actual use of Islamic FinTech services. In turn, actual use is modeled as a central behavioral outcome that directly influences users’ intention to continue using these services. Additionally, service quality and system quality are introduced as moderating variables that condition the relationship between actual use and continuance intention. Specifically, these quality-related factors are expected to strengthen or weaken the extent to which prior usage translates into sustained behavioral intention. This structure reflects a post-adoption perspective, where continued engagement is shaped not only by initial beliefs but also by usage experience and system performance.

## 3. Research methodology

### 3.1 Ethics approval

informed consent was obtained for each participant. They were reassured that their participation is voluntary and that they were free to withdraw at any time. In addition, all information was gathered anonymously and handled confidentially. The study design assured adequate protection of study participants, and neither included clinical data about patients nor configured itself as a clinical trial. The study and questionnaire were presented to the Scientific Research Committee, which established an Ethics Committee that approved proceeding with the research according to the established guidelines. The name of the Committee is (Scientific Research Committee) and It is the committee supervising scientific and academic activities and tasks, and the reference number is (03/09–2025).

A cross-sectional survey design was employed in this study. Data were collected from active users of Islamic FinTech services in Jordan using an online questionnaire. Participants were recruited through social media platforms, professional networks, and direct electronic invitations. To be included in the study, respondents were required to have prior experience using at least one Islamic FinTech service and to reside in Jordan at the time of data collection. This approach ensured that the sample was relevant to the study objectives and reflected actual user experience with Islamic FinTech platforms.

A total of 279 questionnaires were distributed, of which 232 valid responses were retained for analysis, yielding a response rate of approximately 83%. A screening question was included at the beginning of the survey to ensure that respondents had prior experience using Islamic FinTech services. Responses that were incomplete or failed the screening criteria were excluded. In addition, data collection was conducted over a period of 40 days, from 7 July 2025–16 August 2025.

The research constructs were assessed using items derived from relevant literature sources (See Table 1).

**Table 1:** Sources of measurement.

Constructs	References
Perceived Usefulness	[65,66]
Self-efficacy	[67,68]
Subjective norms	[69]
Perceived trust	[70,71]
Actual Use of Islamic FinTech	[9,11]
Behavioral intention to continue use	[72]
Service quality	[73,74]
System quality	[75]

The questionnaire, which includes 24 items, is shown in Table 2.

**Table 2:** Questionnaire items.

Constructs	Code	Statements
Perceived Usefulness	PU1	I find Islamic FinTech services useful for managing my financial transactions effectively.
	PU2	Using Islamic FinTech improves my efficiency in financial management.
	PU3	I believe that Islamic FinTech services add value to my financial activities.
Subjective Norms	SN1	People important to me think that I should use Islamic FinTech services.
	SN2	My family and friends who use Islamic FinTech influence my decision to use it as well.
	SN3	In my community, using Islamic FinTech services is seen as a good financial practice.
Self-efficacy	S-E1	I feel confident using Islamic FinTech services to perform financial transactions.
	S-E2	I can handle any issues that arise while using Islamic FinTech platforms.
	S-E3	My skills are sufficient to use Islamic FinTech services effectively.
Perceived Trust	PT1	I trust the security features of Islamic FinTech services to protect my financial information.
	PT2	I believe that Islamic FinTech companies operate in a transparent and trustworthy manner.
	PT3	My trust in the reliability of Islamic FinTech services influences my usage frequency.
Service Quality	SEQ1	The quality of customer service in Islamic FinTech meets my expectations.
	SEQ2	Islamic FinTech services are consistently reliable and accessible when I need them.
	SEQ3	The responsiveness of support services in Islamic FinTech enhances my user satisfaction.
System Quality	SYQ1	Islamic FinTech systems are always functional and available without technical issues.
	SYQ2	The user interface of Islamic FinTech platforms is easy to navigate and use.
	SYQ3	I am satisfied with the speed and efficiency of Islamic FinTech services.
Actual Use of Islamic FinTech	ACUIF1	I regularly use Islamic FinTech services for my financial needs.
	ACUIF2	My use of Islamic FinTech services has increased over the past year.
	ACUIF3	I prefer using Islamic FinTech services over traditional financial services.
Behavioral Intention to Continue Use	BICU1	I intend to continue using Islamic FinTech services in the future.
	BICU2	I will recommend Islamic FinTech services to others because I plan to keep using them.
	BICU3	My positive experiences with Islamic FinTech services make me want to continue using them.

Participants responded to each question on a Likert scale ranging from 1 (strongly disagree) to 5 (strongly agree). In answer to the question as to whether and to what extent common method bias (CMB) was present in the data collected, [76] suggested a procedure that involves computing the full collinearity variance inflation factor, or FCVIF, across all of the variables in the research model, and this procedure was followed. We found that the greatest FCVIF value was 2.987, which suggested that the measurement model was free of CMB*.*

Table 3 presents the demographic characteristics of the respondents. The sample reflects a diverse group of Islamic FinTech users in terms of gender, age, education level, and usage experience.

**Table 3:** Demographic profile.

Variable	Category	Frequency	Percentage (%)
Gender	Male	128	55.2
	Female	104	44.8
Age	18–25 years	72	31.0
	26–35 years	98	42.2
	36–45 years	42	18.1
	Above 45 years	20	8.7
Education Level	Diploma or below	38	16.4
	Bachelor’s degree	132	56.9
	Master’s degree	48	20.7
	PhD	14	6.0
Usage Experience (Islamic FinTech)	Less than 1 year	46	19.8
	1–3 years	104	44.8
	More than 3 years	82	35.4
Frequency of Use	Occasionally	58	25.0
	Monthly	72	31.0
	Weekly	64	27.6
	Daily	38	16.4

Although the sample size of 232 respondents is adequate for PLS-SEM analysis, potential sampling bias should be acknowledged due to the use of a non-probability convenience sampling approach, which may overrepresent users who are more digitally engaged with Islamic FinTech services. Additionally, respondents were not limited to a specific platform but reported using a variety of Islamic FinTech applications available in Jordan, including mobile wallets, digital payment systems, and Islamic banking apps. This approach allows for capturing general user behavior across Islamic FinTech services; however, it may limit the ability to account for platform-specific differences, which should be considered when interpreting the results.

Participation in the study was voluntary, and all respondents were informed of the study’s purpose prior to completing the questionnaire. Informed consent was obtained electronically at the beginning of the survey. Respondents were assured of anonymity and confidentiality, and no personally identifiable information was collected. The data were used solely for academic research purposes and analyzed in aggregate form to ensure participant privacy.

## 4. Data analysis

The research utilized version 4 of the Smart Partial Least Squares software and applied variance-based Structural Equation Modeling. This software allows for the estimation of a structural model, including the scales’ reliability and validity, before proceeding to the model’s assessment and the testing of hypotheses. The model fit the data well and provided rather clear results [77].

### 4.1 The measurement model assessment

This study began by assessing the quality of the item-construct relationships, which is fundamental to both reliability and validity (see Table 4). The results from PLS confirm that all items had satisfactory factor loadings (≥ 0.60). We also checked the homogeneity of the items to ensure they all measure the same construct, and we used two indices to estimate the likelihood that an arbitrarily selected half of the items would produce the same results as the other half if both sides were tested. One of these indices is well known, and it is called Cronbach’s Alpha (α). The other index is not so well known, and it is called Composite Reliability (CR). Both indices had satisfactory results (≥ 0.70). In addition, to gauge convergent validity, we looked at the Average Variance Extracted (AVE). The AVE helps us understand how much of the variance in a construct’s items is actually due to the construct and not due to the error associated with the measurement instruments. The threshold of acceptability for the AVE is 0.50; anything below it suggests that the variance in the items is not largely a result of the items being good indicators of the underlying construct. The values of the AVE for our four constructs exceeded the threshold and this aligned with [77].

**Table 4:** Measurement model criterion.

*Construct*	*Cronbach’s* *alpha*	*CR*	*AVE*
Actual Use of Islamic FinTech	0.782	0.807	0.696
Behavioral intention to continue use	0.854	0.854	0.774
Perceived Trust	0.771	0.788	0.679
Perceived Usefulness	0.752	0.880	0.631
Self-efficacy	0.797	0.842	0.705
Service quality	0.701	0.715	0.621
Subjective norms	0.817	0.847	0.733
System quality	0.780	0.779	0.695

The [78] criterion was used to assess the discriminant validity. As Table 5 shows, the square root of the AVE for each construct exceeded the correlations with other constructs. Thus, the findings confirm that the discriminant validity meets the Fornell-Larcker criterion.

**Table 5:** Discriminant validity.

No.	Constructs	1	2	3	4	5	6	7	8
1	Actual Use of Islamic FinTech	0.834							
2	Behavioral intention to continue use	0.414	0.879						
3	Perceived Trust	0.679	0.467	0.824					
4	Perceived Usefulness	0.609	0.367	0.518	0.794				
5	Self-efficacy	0.633	0.486	0.624	0.410	0.839			
6	Service quality	0.489	0.390	0.572	0.429	0.410	0.788		
7	Subjective norms	0.679	0.485	0.694	0.555	0.552	0.542	0.856	
8	System quality	0.336	0.514	0.385	0.493	0.353	0.361	0.431	0.834

Discriminant validity was further assessed using the Heterotrait–Monotrait Ratio (HTMT). The results indicated that all HTMT values were below the recommended threshold of 0.90, ranging from 0.418 to 0.848, thereby confirming adequate discriminant validity among the study constructs. These findings demonstrate that each construct is empirically distinct from the others and that multicollinearity issues were not present in the measurement model.

Moreover, Collinearity assessment was conducted using the Variance Inflation Factor (VIF). The results indicated that all VIF values ranged between 1.170 and 2.885, which are below the recommended threshold of 5.0. These findings confirm the absence of multicollinearity issues among the measurement items and demonstrate that collinearity did not threaten the validity of the measurement model.

### 4.2 The structural model

The study’s inner model shows how the present research variables are connected. This helps understand their connections between hidden factors. Table 6 shows the size and importance of these connections by showing Beta values, T-Statistics, and P-Values for path strengths. In this study, the conclusions from all research hypotheses are confirmed by using a significance level of 0.05 with an inner SEM-PLS model [77]. The table shows the route coefficients in Table 6.

**Table 6:** Hypotheses testing.

*H*	*Paths*	*β value*	*T value*	*P-value*	*The result*
*1*	Perceived Usefulness - > Actual Use of Islamic FinTech	0.255	5.001	0.000	*Accepted*
*2*	Self-efficacy - > Actual Use of Islamic FinTech	0.259	4.146	0.000	*Accepted*
*3*	Subjective norms - > Actual Use of Islamic FinTech	0.246	3.383	0.001	*Accepted*
*4*	Perceived Trust - > Actual Use of Islamic FinTech	0.255	5.001	0.000	*Accepted*
*5*	Actual Use of Islamic FinTech - > Behavioral intention to continue use	0.179	2.817	0.005	*Accepted*
*6*	Actual Use of Islamic FinTech - > Service quality - > Behavioral intention to continue use	0.071	1.891	0.059	*Rejected*
*7*	Actual Use of Islamic FinTech - > System quality - > Behavioral intention to continue use	0.131	3.548	0.000	*Accepted*

Bootstrapping was performed on the confidence intervals for each structural relationship so as to determine if they are stable and significant. Each of the confidence intervals had a value greater than zero (i.e., none included zero), thus establishing that there is a statistically significant relationship between each construct. For instance, the confidence interval for Actual Use of Islamic FinTech and Behavior Intention to Continue Using Islamic FinTech ranged from.121 to.290; this confirms the existence of a positive relationship between them. These results suggest the structural model’s estimates were both reliable and robust.

**Fig 2:**
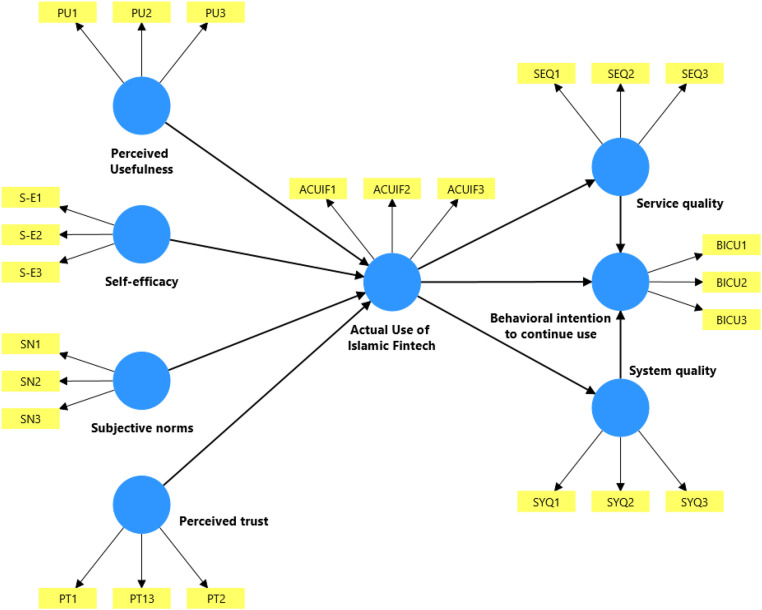
Path coefficient analysis.

In addition, the outcome of Smart PLS ‌‌(Fig 2) indicates that Actual Use of Islamic FinTech, accounts for 63.4% of the variance in actual use of Islamic FinTech services.

Model fit was assessed using several fit indices, including SRMR, d_ULS, d_G, Chi-square, and NFI. The SRMR value for the saturated model was 0.089, which is within the acceptable threshold of 0.10, indicating an acceptable model fit. Additionally, the NFI values of 0.650 (saturated model) and 0.626 (estimated model) suggest a moderate level of model fit, which is considered acceptable in exploratory PLS-SEM research. Overall, the model fit indices indicate that the proposed model demonstrates an acceptable level of fit and predictive adequacy.

Also, the f² effect sizes were calculated to determine how much each external factor contributed to the internal factors. System quality had the largest effect size for continuing to use the system (f² = .194); and also had a large effect size for using the system (f² = .127) compared to service quality (f² = .314). In addition, perceived trust (f² = .116) and self-efficacy (f² = .106) showed some evidence of having small-to-moderate effects on actual use of Islamic Fintech services; as did subjective norms (f² = .074) and perceived usefulness (f² = .053). However, service quality (f² = .023) and actual use (f² = .050) had smaller-than-expected impacts on continuing to use the system.

## 5. Discussion

From a theoretical standpoint, the findings reinforce and extend established behavioral and technology-adoption models by demonstrating that continuance behavior in Islamic FinTech is governed by a distinct post-adoption logic. In contrast to many other TAM- or TPB-based studies which identify behavioral intentions as the primary variable of interest; this study positions actual use as an explanatory factor for user behavior. Additionally, the differing levels of influence by system quality and service quality demonstrate that the success or failure of technology can be a much stronger predictor of sustained use of a product versus support offered through customer service. This is significant because it demonstrates the importance of both of these variables within digitally-enabled financial services in creating ethically grounded environments.

The results need to be evaluated based upon the social and cultural characteristics that exist within the context of the study. In an environment based upon Islamic finance, decisions related to financial matters are not solely determined by the consideration for efficiency; rather they are significantly impacted by religion and culture (social norms) and ethics. A large part of the findings regarding the relationship between perceived utility, norms, and trust and actual usage reflects the group or collective nature of financial activity in these types of environments. For example, there exists a significant amount of evidence which suggests that family influences (e.g., family members who endorse or discourage), community acceptance (endorsement), and perceptions of compliance with Shariah law all impact financial behaviors. As opposed to situations where the user(s) adopt products or services individually, in order for users to continue using Islamic FinTech applications, they must perceive the application as being acceptable to their peers and compliant with the tenets of Islam. Understanding this aspect of the context explains how influential normative and trust-related factors will be throughout the entire process of adoption.

The results supported that perceived usefulness significantly impacts the actual use of Islamic FinTech. This result aligns with prior work locating user confidence and trust as motivators in facilitating innovative finance solution adoption [5,48]. It also supports prior research prioritizing Shariah conformance, transparency, and simplicity in fostering favorable perceptions towards Islamic FinTech services [9]. Additionally, [17] indicates users that discover Islamic FinTech sites as useful and trustworthy will most likely develop towards non-conventional systems in banks. Adoption diffusion operating through information technologies, improving perceived usefulness, has been found as surmounting access barriers [18], and user-oriented interfaces reflect this perception correspondingly [11]. Additionally, mobile technology has multi-dimensional advantages fostering wider acceptability and institutional inclusions [19].

The study confirms that subjective norm plays a significant role in using Islamic FinTech. This is in alignment with what has been referenced by [21] referenced the religious and social pressures regarding the adoption of technological advancements when it comes to Islamic finance. What has been referenced by [22] is also interesting in that there has been critical dependence on positive endorsements by most revered religious leaders as being pivotal towards enhanced adoption regarding Islamic FinTech. Likewise, [5,24] have also stated that in collectivistic society where there has been enhanced social control, there shall be enhanced adoption regarding Islamic FinTech. On social media, what has been stated by [20] has been regarding social media facilitating the enhancement of the objective norm by means of eliciting the discourses which place in the limelight the support and following regarding Islamic FinTech in line with the tenets of Islamic Finance [79,80].

This study found that self-efficacy plays a pivotal role in the actual use of Islamic FinTech, which confirms that people’s beliefs about their capabilities to use technology fosters its acceptance. These results align with prior works like those from [7], who argued that self-efficacy affects how people’s confidence translate to adoption of Islamic FinTech platforms. [27] have further pointed out that self-efficacious persons are bound to accept Islamic FinTech services because of the confidence they have in using the platforms. Furthermore, [28] showed that users with mastery of electronic transactions tend to shift from traditional to Islamic FinTech services. Also, [29] noted that self-efficacy results in favorable intentions and actions concerning utilizing services. For [30], self-efficacy lessens fears surrounding technology and allows for more engagement with Islamic FinTech applications. Similarly, [31] detail how certain self-efficacy construct enhancing specialized literacy programs result in technology use because of the increased self-efficacy.

Trust greatly affects the actual use of Islamic Fintech services [19]. This is consistent with other studies that also report that trust in Islamic financial service providers (particularly in Sharia compliant, transparent, honest, and reliable providers) contributes to users’ movement from mere intentions to actual usage [35–36]. Other studies also report convergence with the findings of the current study to the extent that perceived trust in fraud, data security, ethics, and other forms of policy and procedure manipulation of Islamic Fintech services is likely to reduce users’ apprehensiveness as to willingness to use Islamic Fintech services [37–39]. Furthermore, it is common to assume that users do not only develop intentions, but do possess active behavioral engagement to the extent that they trust the credibility of the institutions as well as their adherence to regulations and the standards of service delivery; and do perceive Islamic Fintech services as dependable alternatives to conventional banking [40–42].

The findings show that the actual adoption of Islamic FinTech significantly affects users’ intention to continue using those platforms. These findings corroborate earlier studies that indicated users having Islamic FinTech platforms interactions tend to continue using those platforms. [56] noted that the perception of ease when using the platforms positively influenced users’ continued usage of the platforms. Similarly, [26] recognized reliability as the foremost factor contributing to users’ intention to continue using Islamic FinTech services. [57,81] also showed that the ease of Islamic FinTech services due to the simplicity of the transactions, compliance of the services to Shariah, and the quality of the services positively influenced the intention to use Islamic FinTech services long-term. Also, [58] proposed that the lack of trust in the system diminishes with the system’s use, and with that, the system’s use is encouraged. [60,61] studied the factors of Islamic FinTech program effectiveness, secure payment channels, personalization, and user familiarity on the retention of Islamic FinTech users [74,82].

Unlike earlier studies which demonstrated the importance of service quality on the increased use and ongoing utilization of Islamic FinTech platforms, this study demonstrated a lack of service quality on the behavioral intent to perpetually use Islamic FinTech. This gap can be attributed to a number of reasons. First, even though previous studies such as [43,82] described the importance of service quality on satisfaction and loyalty, the actual service experience on the quality of technical support and customer service, as discussed by [47,83], leaves a lot to be desired. Non-optimized users suffering from technical difficulties and poor customer service are not likely to view such support as valuable enough to increase their intent to use the service. Also, [48–49] posited that service quality increases trust and improves customization of services to user demands, increasing the likelihood of continuing engagement. However, if these users are deprived of these attributes which lead to enhancement of their overall service experience, the expected positive moderation is likely to be offset, hence the lack of these findings.

The outcomes confirmed that system quality moderates the link between the actual use of Islamic FinTech and a person’s behavioral intention to continue using it. This supports the assertions that emphasize the effect of strong system performance on user participation in Islamic FinTech platforms engagement [51–52]. It also supports the previous studies which noted that dependable, safe, and user-friendly systems provide digital trust and increase participation [5,55]. Furthermore, certain studies have found that the system quality attributes of rapid processing, low response time, and well- organized graphics are crucial in transforming satisfaction of first-time users into long-term intentions of behavioral engagement [53]. Nonetheless, experts warn that high rates of technical failures and security violations create suspicion and reduce the odds of continued use [28,54].

From an institutional perspective, the findings also reflect the role of regulatory frameworks and digital infrastructure in shaping continued use behavior. In emerging Islamic FinTech markets such as Jordan, institutional trust is closely linked to perceptions of regulatory oversight, system security, and platform reliability. The significant moderating role of system quality suggests that users place greater emphasis on technical robustness and operational stability once initial adoption has occurred. This may be attributed to heightened sensitivity toward system failures or security breaches in financial applications, particularly in contexts where regulatory credibility and technological maturity are still evolving. Consequently, system quality becomes a critical mechanism through which actual use translates into sustained behavioral intention.

Beyond aligning with prior empirical findings, the results of this study provide deeper theoretical insight into post-adoption behavior in Islamic FinTech contexts. Specifically, the findings suggest that actual use functions as a central behavioral mechanism through which cognitive, social, and trust-related factors are translated into sustained engagement, extending traditional intention-based models such as TAM and TPB. This highlights a shift from intention-driven adoption toward experience-based reinforcement, where repeated usage becomes a stronger predictor of continuance intention than initial perceptions alone. Moreover, the non-significant moderating effect of service quality, contrary to expectations, indicates that users may prioritize system functionality and reliability over service interactions once familiarity with the platform is established. This finding suggests that, in digital financial environments, technical performance may outweigh human-centered service elements in shaping long-term usage behavior. Collectively, these insights contribute to refining existing theoretical frameworks by emphasizing the role of usage experience and system-centric factors in explaining sustained Islamic FinTech adoption.

## 6. Implications and future research

### 6.1 Theoretical implications

This research expands the theoretical understanding of both Islamic Finance and Fintech by identifying the factors that contribute to continued usage of Islamic FinTech Services. While previous literature has focused primarily on users’ decision to adopt new technological services (initial usage), it is typically at the expense of the factors that continue to drive users to utilize such technologies after their initial usage. This study bridges this knowledge gap through an integration of five constructs; service quality, perceived usefulness, subjective norms, self-efficacy, and perceived trust into a single model, which extends the applicability of two well-established theories (Theory of Planned Behavior and Diffusion of Innovations) within the Islamic FinTech context.

Theoretically, this research extends the Islamic FinTech literature through the application of TPB and DOI in the context of continuance behavior versus initial adoption. A key contribution is the representation of actual use (AU) as an important post-adoptive behavioral mechanism that links cognitive, social and trust-based constructs with continued use. An additional contribution includes the differentiation of the role of both service quality (SQ) and systems quality (SYQ) by representing SQ and SYQ as moderating influences instead of direct precursors. As such, this study provides a more detailed description of how experience with technology and service quality will influence long-term continued use of Islamic FinTech.

The results show how subjective norms and self-efficacy can further increase the perceived value of utilizing Islamic FinTech for customers increasing their likelihood of continuously using these technologies thus providing additional insights into customer behavioral patterns when adopting new technologies.

### 6.2 Practical implications

The study’s results show that all five variables (i.e., system performance, reliability, perceived usefulness, trust, and self-efficacy) have a positive association with continued use in the Islamic FinTech context. Additionally, the results suggest that system quality has a moderating effect on the relationship between actual usage and continuation intent; that is, technical functionality and operational stability can affect how the relationship between the two constructs are expressed. Therefore, this research provides an empirical basis for studying post-adoption behaviors in the Islamic FinTech context, as well as an improved understanding of what contributes to users’ sustained engagement in digital financial services.

### 6.3 Limitations and future research

The present study is based on data collected from one location Jordan. Therefore, the study’s results will likely suffer from insufficient external validity (i.e., the results might not generalize to different cultures, economies and regulatory conditions of Islamic banking in other countries). We encourage future studies to consider greater geographically-based samples so that we can achieve a more complete understanding of the Islamic FinTech ecosystem. Further, this study depends on responses to a self-reporting instrument (questionnaire), therefore it may be subject to both social desirability bias and common method bias. Common method bias is the “error” resulting from respondents providing an answer they think is the socially correct response instead of accurately reflecting their actual views and actions.

Also, since the researchers used a nonprobability sampling strategy (convenience sampling) for this study, this reduces the potential for generalizing the results of the study beyond those who were studied. For example, because the sample was mostly made up of people who use digital technology regularly when using Islamic financial technology services, the results may reflect a higher degree of participation among those who are technology savvy.

Finally, the study used a cross-sectional design to assess relationships at one point in time; therefore, it does not allow us to observe changes in user beliefs or behavior patterns over time. Longitudinal and qualitative designs could provide additional insights into sustained adoption and continued use of Islamic financial technology services. Other studies could investigate how emerging technologies like artificial intelligence and blockchain relate to trust and security issues and regulatory considerations in the context of Islamic financial technology.

## 7. Conclusion

With the concomitantly experienced IT revolution globally, Islamic FinTech (IFT) is contributing factor in Islamic Financial Institutions (IFIs) by evoking inclusion, ethical finance, and tech-savviness. It was the primary goal in this study to bridge the literature gap through the assessment of service quality, subjective norm, self-efficacy, and perceived trust in ongoing use in IFT. Data are surveyed by a structured questionnaire that explicitly depicts the importance of factors in user satisfaction and ongoing use in Islamic FinTech services. User satisfaction was not found to be significantly dependent on service quality in this study, suggesting that its role in influencing continued use of Islamic FinTech services may be limited or context-dependent. However, it has been confirmed that subjective norms and self-efficacy act as key drivers of user behavior toward the utilization of the technology, reflecting the importance of social influence and individuals’ confidence in their ability to use Islamic FinTech services. Also, it was demonstrated that compared to others, ease and safety were provided through trust in the utilization of the facility thus facilitating the users in adopting and ongoing utilization of the technology.

This study expands the knowledge of Islamic FinTech continued usage behavior through investigating the associations between perceived useful, subjective norms, self-efficacy, perceived trust, service quality, system quality and continuance intention. Findings show that both cognitive (i.e., perceived usefulness), social (i.e., subjective norms) and trust related factors are significant to usage behavior; however, system quality is influential for the association between usage behavior and continued usage. Additionally, this study has extended prior studies focused upon Islamic FinTech, as it examines post adoption behavior within a specific Jordanian context. Future studies could explore how regulatory changes, technological advancements and differences in the markets environment will continue to shape long term Islamic FinTech usage.
